# A Capacity Building Program to Improve the Self-Efficacy of Key Workers to Support the Well-Being of Parents of a Child With a Disability Accessing an Early Childhood Intervention Service: Protocol for a Stepped-Wedge Design Trial

**DOI:** 10.2196/12531

**Published:** 2019-04-03

**Authors:** Elise Davis, Dana Young, Kim-Michelle Gilson, John Reynolds, Rob Carter, Utsana Tonmukayakul, Katrina Williams, Lisa Gibbs, Rachael McDonald, Dinah Reddihough, Jane Tracy, Jennifer Morgan, Paul Ireland, Cassie Kenyon, Rod Carracher

**Affiliations:** 1 Jack Brockhoff Child Health and Wellbeing Program Centre for Health Equity, Melbourne School of Population and Global Health University of Melbourne Carlton Australia; 2 Mind Australia Limited Heidelberg Australia; 3 Neurodevelopment and Disability The Royal Children's Hospital Parkville Australia; 4 School of Public Health and Preventive Medicine Faculty of Medicine, Nursing and Health Sciences Monash University Melbourne Australia; 5 Deakin Health Economics Centre for Population Health Research, School of Health and Social Development, Faculty of Health Deakin University Geelong Australia; 6 Developmental Disability and Rehabilitation Research Murdoch Children’s Research Institute Parkville Australia; 7 Department of Paediatrics University of Melbourne Parkville Australia; 8 Department of Health and Medical Sciences Faculty of Health, Arts and Design Swinburne University Hawthorn Australia; 9 Centre for Developmental Disability Health Monash Health Clayton Australia; 10 Yooralla Melbourne Australia

**Keywords:** mental health, early intervention, allied health, health services for persons with disabilities, capacity building, cost analysis

## Abstract

**Background:**

Early childhood intervention services support children with disabilities or developmental delays from birth to school entry with the aim to achieve optimal outcomes for children and their families. A transdisciplinary approach to delivering early childhood intervention, particularly the key worker model, is considered the best practice, where allied health professionals (eg, speech pathologists, physiotherapists, occupational therapists, psychologists, and special educators) and the family work together as a collaborative team to share information, knowledge, and skills across disciplinary boundaries, with a key worker coordinating and delivering most of the intervention to achieve the goals for the child and their family. Initial qualitative research demonstrated parents want their key worker to also support their mental well-being. Poor mental well-being of parents of a child with a disability is of relevance to key workers because of its association with poor child-related outcomes. One of the major challenges key workers report in supporting families is managing parent distress and, because of lack of confidence, is a secondary negative impact on their own well-being.

**Objective:**

This trial has been developed in response to the negative cycle of low professional confidence to support parents’ mental health, increased key worker stress, and high turnover of employees working within a disability service setting.

**Methods:**

A stepped-wedge design is used to deliver and evaluate a capacity building intervention program, over a 9-month period, for key workers to improve both parent and staff mental well-being. The primary outcome is key workers’ self-efficacy in supporting parental mental well-being. Secondary outcomes include manager self-efficacy in supporting key workers and staff perceptions of supervisory support, staff job-related mental well-being, parental satisfaction with their key worker, parental mental well-being, and cost-consequence of the program.

**Results:**

This study was funded in October 2014, supported by an Australian National Health and Medical Research Council Partnership Project grant (Grant number 1076861). Focus groups and individual face-to-face interviews were conducted from February to November 2015 with 40 parents who have a child with a disability and 13 key workers to gain insight into how the disability service could better promote child and family health and well-being and to inform about the development of the trial.

**Conclusions:**

The stepped-wedge study design is practical and ethical for research with a vulnerable population group of parents of a child with a disability, providing high quality data with all participants exposed to the intervention by the end of the trial.

**Trial Registration:**

Australian New Zealand Clinical Trials Registry ACTRN12617001530314; https://www.anzctr.org.au/Trial/Registration/TrialReview.aspx?id=372578 (Archived by WebCite at http://www.webcitation.org/76XjDavnG)

**International Registered Report Identifier (IRRID):**

DERR1-10.2196/12531

## Introduction

### Background

In 2015, 4.3 million Australians were reported as having a disability, including 327,400 children (7.6%) aged 0 to 14 years [1]. Early Childhood Intervention Services (ECISs) are an Australian government–funded service that supports children with disabilities or developmental delays from birth to school entry and their families [2]. These services aim to provide families with the knowledge, skills, and support to meet the needs of their child and to optimize their child’s development and participation in family and community life.

A transdisciplinary approach to delivering early childhood intervention, particularly the key worker model, is considered the best practice, where allied health professionals (eg, speech pathologists, physiotherapists, occupational therapists, psychologists, and special educators) and the family work together as a collaborative team to share information, knowledge, and skills across disciplinary boundaries, with a key worker coordinating and delivering most of the intervention to achieve the goals for the child and their family [3-5].

### Theoretical Framework

Family-centered practice (FCP) is important to the delivery of care in ECISs. This is a broadly defined philosophy which places families in central and pivotal roles in decisions and actions involving the child, parent, and family priorities and preferences [6]. Implicit in the philosophy of FCP is the need for services to be responsive to the family situation and to mobilize support that can produce optimal child, parent, and family benefits [7,8]. FCP and the key worker model have been linked with increased parent satisfaction, decreased parent stress, and improved child outcomes [9-14]. However, applying FCP principles has been difficult with the criticism of it being espoused rather than enacted in everyday practice and areas being deemed important for families and professionals varying slightly [15-17].

Research on the mental well-being of parents of children with a disability is of particular relevance to FCP within ECISs. Mental well-being is defined as “a dynamic state that refers to individuals’ ability to develop their potential, work productively and creatively, build strong and positive relationships with others, and contribute to their community” [18]. Several aspects of parental mental well-being can be adversely impacted when caring for a child with a disability, including mental and physical health, marital relationships, and participation in social and economic life [19-26]. Poor mental well-being is of relevance to key workers within ECISs because it is a significant risk factor for poor child-related outcomes [27-30]. Furthermore, given that the principles of FCP acknowledge the needs of the family holistically, parental mental well-being is of high relevance to ECISs. However, in the few research studies available, those that have investigated support for parental mental well-being suggest that disability services for children do not adequately accomplish this [31-34]. Our own data also support this. A recent review found limited training or support for staff in transdisciplinary teams to work confidently outside their disciplinary boundaries, with this issue needing to be addressed for the model to truly be considered best practice [35]. For the transdisciplinary model to work well for families, the professional competencies of the key worker go beyond discipline-specific knowledge and include personal qualities such as empathy, sensitivity, listening effectively, interpersonal communication skills, and interacting with authenticity [5].

### Setting

This research study was codeveloped by a cross-sectoral team of academics and clinicians in partnership with a major Victorian nongovernment disability service provider in Victoria, Australia, and included an initial qualitative scoping study to identify what children with a disability and their families required to optimize their health and well-being and how staff can facilitate this through the service. The disability service provider offers a wide range of support services to people of all ages who either are born with or acquire a disability. The *Pursuit of Wellbeing* program involves staff within their ECIS, which provides services to children aged up to 6 years, who vary in their severity of disability or developmental delay, family circumstances, and cultural background. The service has 6 different ECIS sites across metropolitan Melbourne which for the purpose of this study are known as *hubs*. They are geographically spread, approximately 25 kilometers apart, to service the suburban population. Each family has a key worker assigned to them from a transdisciplinary team of allied health professionals. Across the 6 hubs, there are approximately 60 key workers supporting 600 families. The disability service provider will implement the program across the 6 hubs using a phased process over a 9-month period, and all key workers and managers will receive the program as part of the trial, which is supported by the executive management team at the service.

### Phase 1: Identification of Family and Staff Needs

In the first phase of development of this trial, a combination of focus groups and individual face-to-face interviews were conducted with 40 parents who have a child with a disability and 13 key workers to gain insight into how the disability service could better promote child and family health and well-being. Parents reported they were satisfied with the professional advice and support that their child received; however, they felt that they did not receive adequate support for their own mental well-being [36]. Concurrently, key workers also reported that one of their major challenges was managing the high rates of parental distress and their need for greater confidence and skills in supporting parental mental well-being. They did not feel confident to refer parents to relevant support services as key workers were unclear whether addressing parents’ mental well-being during a home visit to the family fell within the boundaries of their role, prioritizing support for the child only, an additional barrier being the lack of knowledge of local services to support parents of a child with a disability. The staff also identified that it would be useful for each team to have access to a key worker with a background in psychology on the team. Ideally, key workers with a psychology background would be available within all teams, but this is dependent on successful recruitment to these positions by the organization.

### Phase 2: Development of the Pursuit of Wellbeing Program

A new capacity building program, titled *The Pursuit of Wellbeing*, was then codeveloped based on the findings identified in phase 1 of the research and the current literature, with the aim to build the self-efficacy of key workers to better support the mental health and well-being of parents (Textbox 1). A capacity building framework was selected as it encompasses actions aimed at strengthening the skills and capabilities of the individuals, organization, systems, and wider community [37,38]. Increased capacity at the individual level is likely to increase the self-efficacy of key workers. Self-efficacy is an aspect of empowerment relating to how people perceive their ability to manage challenging situations and accomplish goals. By building the self-efficacy of key workers to support parental mental well-being, we anticipate their own well-being will be impacted by increasing their confidence in how to manage challenging situations and providing a sense of personal accomplishment in their service to the parents and carers [39]. This is important given that high staff stress and poor morale has been linked to burnout, absenteeism, and high staff turnover [40,41], and critically, these factors may in turn result in a lack of support for parents. This trial thus seeks to disrupt the current negative cycle of low professional confidence, increased stress and high turnover of employees, and continuing unmet needs of parents [24,42].

### Phase 3: Delivery of the Pursuit of Wellbeing Program

The intervention program will be implemented by a disability service provider as part of an organizational system change involving all key workers and managers providing an ECIS to families with children aged 0 to 6 years with a disability or developmental delay. The training will be facilitated by an internal senior manager who is also a clinical psychologist. An internal position was chosen not only because of their understanding of the intricacies of mental health promotion but also to tailor the program to suit the organizations needs and embed the program into existing organizational operations. The training is designed to include educational modules, discussion, and provision of a toolkit of psychological resources to support key workers in discussing well-being with parents and for managers to provide well-being support to their staff. Managers of each hub will then assume responsibility for the ongoing implementation of the program to their staff, with ongoing support and advice available from the facilitator. In this way, the program, if effective, will be sustainable. This protocol outlines the methodology of the evaluation of the *Pursuit of Wellbeing* program undertaken by the research team.

**The *Pursuit of Wellbeing* capacity building program to support staff efficacy in managing the mental wellbeing of parents in an early childhood intervention service.**

Training program

- Key workers and managers
  - Module 1: Strengthening capacity to support parental mental wellbeing. Topics covered include: Importance of family-centered practice to promote wellbeing of child with a disability; How to open up conversation around parental wellbeing; Identifying red flags for poor mental health; Identifying coping types; How to refer to appropriate supports.
  - Module 2: Staff wellbeing and role boundaries. Topics covered include: How to identify and manage stress in the workplace; Self-care strategies; Outline of key worker roles, responsibilities and boundaries, and balancing the role; Available team and organizational support strategies, such as promoting debriefing, and counseling options if staff are experiencing heightened stress or distress.
- Managers
  - Module 1: Strengthening support for key workers. Topics covered include: How to identify, support, and manage stress in the workplace (in others and in self); Clarification of key worker role boundaries; How to debrief with staff; How to promote staff self-care. It is anticipated that managers will be a key resource for staff seeking expertise in addressing parental mental well-being. Managers will also discuss the support required for a senior staff member in their team acting as parental well-being champion (further details below). This role will likely be assumed by a key worker appointed also as a team leader position.

Resources

- For key workers and managers
  - The following are provided as hard copies and Web-based via the staff intranet after completion of the study: Organisational Practice Framework incorporating the key messages of the training; Toolkit of positive psychology activities and resources; Tailored referral pathway of local support services; Well-being for parents and carers resource [43].
  - A formalized debriefing process for staff includes: Building the capacity of managers to improve their approach to debriefing during monthly supervision meetings; Developing of a flow chart for staff to know their immediate options to access debriefing and counseling.
  - The appointment of a senior staff member in each team to act as a parental well-being champion. Additional training will be provided to support the champion in their conversations with key workers on supporting parental mental well-being.
- For parents
  - The following are provided as hard copies: Tailored referral pathway of local support services; Well-being for parents and carers resource [43].

## Methods

### Objectives

The objective of this study is to trial a capacity building program that aims to increase the self-efficacy of key workers to support parental mental wellbeing. The research questions are outlined in Textbox 2.

**Figure 1:**
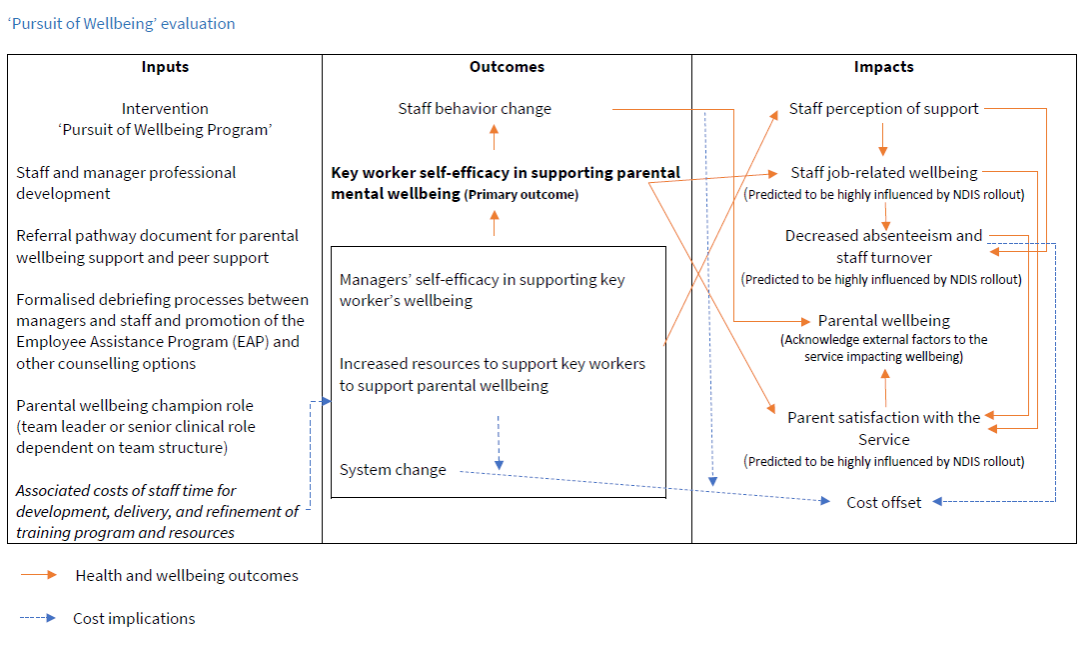
Program logic.

The predicted outcomes and impacts of the intervention are presented in a logic model (Figure 1).

### Trial Design

This study employs a stepped-wedge design to evaluate the program. The design was chosen so that each hub would eventually receive the program and be provided with the new professional development opportunity. The intervention will be rolled out progressively over 9 months. The clusters for randomization will be the hubs (N=6). Randomization will be conducted by a statistician who is independent of the administration of the intervention [44]. The first 2 hubs will not be randomly selected, as senior management advised against this because of the large amount of ongoing organizational change at the service. These 2 hubs will be selected based on readiness to undergo the trial and will receive the program immediately after baseline is established with the remaining 4 hubs randomized 2 at a time, every 3 months thereafter. In this way, hubs that were previously acting as control hubs will progressively receive the program. All hubs are assessed at baseline and every 3 months thereafter. The stepped-wedge design is suitable for a phased evaluation approach such as this, in which there is an imperative to allow all participants to have access to the new program. The design also allows each hub or cluster to act as its own *pre versus post* control and, in the first 2 periods, there are at least 2 hubs acting as controls and at least 2 hubs receiving the program (Table 1). The statistical analysis (see below) will combine the *between-* and *within-* hub information on the effect of the program. We seek to minimize potential bias by not revealing to the key workers when the new program is scheduled to be allocated to their hub. However, this will be self-evident to the key workers in the last 2 hubs. Key workers that operate across multiple sites will be requested to refrain from sharing learnings or materials from the training with fellow staff at other sites who have not received the intervention. Although there is a risk of contamination, this is not anticipated to have a large effect as the number of key workers that work at multiple sites is very small.

**Research questions.**

1. Does the capacity building program increase:
  - Key workers’ self-efficacy in supporting parental mental well-being (primary outcome);
  - Managers’ self-efficacy in supporting staff and parental well-being;
  - Parental mental well-being;
  - Key workers’ and managers’ job-related wellbeing and mental wellbeing;
  - Parental satisfaction with the service provided by their key worker; and/or
  - Key workers’ and managers’ perceptions of supervisory support?
2. Are the program and evaluation methodologies appropriate and feasible for key workers, managers, and parents?
3. Is the program good value-for-money?
4. Is it affordable?

The cost of the capacity building program will be estimated to help determine whether this program is *value-for-money*. Simple dominance would establish this—that is, compared with current practice, is it cheaper with improved effectiveness or cost neutral with improved effectiveness. Similarly, on the flip side, if the program is dominated, then it is not value-for-money—that is, more expensive and less effective or more expensive with no improvement in effectiveness. If the program is both more expensive and more effective, then more complex tests of efficiency are required that establish value-for-money by reference to established guidelines using a metric that enables comparison across alternate uses for limited budgets (eg, a return on investment >5% and a cost per quality-adjusted life-year <AUD$50,000). Value-for-money considerations, together with affordability considerations, will inform the program’s potential to be scaled-up and adopted by other disability organizations.

**Table 1:** The stepped-wedge design (partially randomized).

Hub	Baseline (0 months)	Period 1	3 months	Period 2	6 months	Period 3	9 months
1	X^a^	I^b^	X^c^	I^c^	X^c^	I^c^	X^c^
2	X	I^c^	X^c^	I^c^	X^c^	I^c^	X^c^
3	X	C^d^	X	I^c^	X^c^	I^c^	X^c^
4	X	C	X	I^c^	X^c^	I^c^	X^c^
5	X	C	X	C	X	I^c^	X^c^
6	X	C	X	C	X	I^c^	X^c^

^a^X: assessment.

^b^I: intervention (ie, the new program).

^c^Hub is receiving the program.

^d^C: control.

### Participants and Recruitment

All key workers and managers at each hub have the opportunity to participate in the intervention and this will be strongly encouraged by the management. All key workers and managers at each hub, and families that they support, are eligible and will be invited to participate in the evaluation. To recruit staff, the researchers will attend a staff meeting to talk about the study and commitments involved. Staff will be given a plain language statement and consent form. The manager of each hub will be blinded to which key workers are taking part in the evaluation of the trial to allow key workers to feel comfortable in providing feedback on their current management and any adverse experiences they may have had. Reminder emails will be sent to managers to distribute to all staff to enhance recruitment until the intervention begins. To recruit parents, all key workers, regardless of their involvement in the evaluation, will be requested to give the plain language statement and consent form to parents in their regular fortnightly visits. Many parents do not act immediately on this type of survey because of their day-to-day pressures. To enhance recruitment and reach all parents, key workers will remind each parent in subsequent visits, email reminders will be distributed by an administrator at each site, and the information sheets will be left at the entrance of each service. If a parent requires an interpreter, this can be arranged both to explain the study and, if they consent, to assist them to complete the questionnaires. Parents will be informed and reassured that the service their child receives will not in any way be impacted by their participation in this evaluation. All participants will return their signed consent form directly to the researchers.

### Data Collection

A mixed-methods approach will be employed to gain an understanding of the process and outcome of delivering this program. Researchers will send the consenting participants a link to the online survey at baseline and each 3-monthly time point. Online surveys will be used to collect quantitative data from all consenting key workers and managers to assess their confidence in supporting parental mental well-being, their perceptions of supervisory support, their work-related well-being, and their own mental well-being. Online surveys will also be sent to all consenting parents to assess their mental well-being, their satisfaction with their key worker, and health care resource use. Paper-based surveys were offered as an alternative for parents. Survey data will be collected at baseline and every 3 months for 9 months to reassess these variables pre- and postdelivery of the program. The survey data collected across the intervals will provide information on the program’s impacts and the practical contribution of the program to the service. Survey data will be collected and managed using Research Electronic Data Capture (REDCap) tools hosted at the University of Melbourne [45]. Reminders to complete the online survey will be sent up to 4 times to all participants via email generated by the REDCap program.

Qualitative data will be collected by conducting focus groups at the 9-month time point. A total of 1 focus group at each hub (N=6) will be conducted with key workers to explore their experience with the program, discussing their perceptions about the usefulness of the program and any challenges they faced implementing their learnings and strategies with parents post training. In addition, 1 focus group at each hub (N=6) will also be conducted with parents to explore the impact of the program on their interaction with their key worker and their mental well-being. Semistructured interviews will be conducted with parents if focus groups are unable to be organized. Moreover, 1 focus group will be conducted with hub managers from the different sites to explore their view on the impact of the program on their staff, clients, and on their management role.

### Outcomes

#### Primary Outcome

##### Key Workers’ Self-Efficacy in Supporting Parental Mental Well-Being

We have developed a set of items to measure how confident key workers are in supporting parental mental well-being. A total of 10 items measured on a visual analogue scale (VAS; Multimedia Appendix 1) assess perceived confidence in understanding and supporting parental mental well-being; their knowledge about how to support parents’ well-being and refer parents for additional help; and to communicate with parents about their well-being based on Bandura’s [46] recommendation that self-efficacy questionnaires are task-specific. The items tapping the same domain of efficacy will be correlated with each other and averaged. The primary analysis will be conducted on the average, with each item given equal importance for each individual at each assessment, of the 10 VAS measurements denoting key worker confidence (KWC).

#### Secondary Outcomes

##### Managers’ Self-Efficacy in Supporting Key Workers’ Well-Being

We also developed a set of items, using the scale recommended by Bandura, to measure the perceived confidence of managers in supporting key workers’ well-being in the workplace and to support their staff to adequately support parental mental well-being (Multimedia Appendix 1). A total of 10 items measured on a VAS assess their perceived confidence in understanding, identifying, and supporting staff well-being and workplace stress and in initiative debriefing after a potentially stressful workplace event. The items tapping the same domain of efficacy will be correlated with each other, summed, and averaged. The primary analysis will be conducted on the average, with each item given equal importance for each individual at each assessment, of the 10 VAS measurements denoting manager confidence.

##### Parental Mental Well-Being

Parental mental well-being will be measured using the shortened Warwick-Edinburgh Mental Wellbeing Scale (WEMWBS; Multimedia Appendix 2). The WEMWBS, although less sensitive, has been shown to be reliable and valid [47], and the short 7-item version of WEMWBS was found to satisfy the strict unidimensionality expectations of the Rasch model and be largely free of bias [48]. This scale differs from other scales of mental health in that it covers only positive aspects of mental health, which was an important factor in choosing this scale to assess mental well-being in this vulnerable population. The scale uses a 5-point Likert scale and scores ranging from 1 to 5 for each item will be summed and averaged.

##### Key workers’ and Managers’ Job-Related Well-Being

Job related well-being will be measured using 2 scales as reported by Warr [49,50] (Multimedia Appendix 1).

###### Job-Related Affective Well-Being Scale

The first instrument measures job-related affective well-being using the Institute of Work Psychology Multi-Affect Indicator and includes 12 items. The staff are questioned using a 6-point Likert scale of agreement about how their job has made them feel across the multiple domains. Domain scores are provided on job related anxiety-contentment and depression-enthusiasm; this 2-dimensional model of job-related affects provides a more comprehensive view of emotional states at work than current measures of job satisfaction [51]. This scale reported acceptable internal consistency with a coefficient alpha of .78 [49].

###### Job-Related Mental Well-Being

For the second scale assessing job-related mental well-being, 9 items were selected from 2 out of 5 domains within the scale. These items were selected based on relevance to the intervention to assess self-reported job competence and negative job carry-over for staff. Excluded domains were deemed not appropriate for this evaluation. The scores between the 2 measures will be correlated, higher scores indicating greater contentment and enthusiasm as well as greater competence and negative carry-over.

##### Parent Satisfaction With Key Workers

New items were developed for this study to assess parental satisfaction with the service provided by the key workers, based on previous assessments utilized by Yooralla (Multimedia Appendix 2). A total of 5 items assess parents’ perception of support from their key worker for their social and emotional well-being and their degree of overall satisfaction with the service received from the disability provider.

##### Key Workers’ and Managers’ Perceptions of Supervisory Support

Supervisory support will be measured using some of the high-loading items of the Perceived Supervisor Support scale [52,53]. The Perceived Supervisor Support scale has 8 items based on perceptions of value and support from supervisors. A total of 3 items were chosen to assess perceived support regarding staff goals and help with problems and well-being, with most items from the scale deemed inappropriate for this evaluation. It has a high internal reliability coefficient (0.88) and has been widely used in research, including a recent project examining burnout in therapists working with children with autism [54]. These items will be averaged with each item given equal weighting.

### Process Evaluation

Process data will be collected from staff at each 3-month time point via the online survey to assess attendance, usefulness, and uptake of the intervention components, such as support-seeking behavior and use of the toolkit resources provided. Researchers will also collect data on the fidelity of the training via observation and meetings with the trainer. In addition, the focus groups being conducted at 9 months postintervention with key workers, managers, and parents will provide an opportunity to explore barriers and facilitators to the delivery of the training and translation of the skills learnt within the training to current practice. Any unanticipated outcomes will be discussed in the focus groups with staff.

### Costing of the Intervention

Cost data will be nested within the program and will be closely coordinated with the purpose and data collection of the study. The primary perspective will be that of the service provider, but health sector and client impacts will also be assessed. Cost analysis of the program, comparator, and any cost offsets will be measured based on service activity and resources required for the services to take place. Program cost will reflect the service provider’s accounting practice, for example, cost category and routine cost data collection. A template to standardize the cost data collection will be completed by each hub manager in consultation with the finance manager at baseline and repeated at 3, 6, and 9 months later. Cost offsets refer to the potential for improvement in staff productivity and/or the service provider’s revenue. Staff productivity includes staff turnover rates, absenteeism, and associated implications. The absenteeism and turnover rates can be obtained from the service provider’s records, whereas self-reported absenteeism will be measured using the short version of the World Health Organization Health and Performance Questionnaire [55,56], which will be used in conjunction with staff well-being assessment questionnaires. In addition to the key worker economic-related outcomes, the economic-related implications of parent well-being on health care system utilization will also be quantified by collecting health system resource use.

### Sample Size

The sample size (6 hubs) is pragmatically determined by the number of hubs at the service and the roll-out period for the intervention. We expect approximately 8 out of 10 key workers located at each hub to consent to be repeatedly assessed (at 0, 3, 6, and 9 months) following the start of the study and approximately 3 out of the 10 families that they each support to consent to be surveyed during the study. The effect of the pragmatically determined sample size on the power of the study was investigated as follows. With 6 hubs randomized 2 at a time every 3 months starting after baseline (0 months), we have a replicated (at the cluster or hub level) stepped-wedge design with 3 periods and we have made provision for between 6 and 8 key workers in each cluster to be repeatedly assessed across the periods. Accordingly, we expect to have between 144 and 192 observations of the primary outcome variable (KWC). KWC is measured on a bounded continuous outcome scale from 0 to 100. As a contingency, we assume that approximately 10% of the variance of a measurement on a randomly selected adult is *between-hub* variance and the remaining component of the variance of a randomly selected adult splits into *between* and *within* individual variance subcomponents according to either of the 2 scenarios—an *optimistic* intraclass correlation (ICC) of 0.5 or a *less optimistic* ICC of 0.25. As an example, the baseline mean for KWC may be 70, the total variance may be 18, and the variance components for hubs, individuals, and assessments within individuals may be 2, 4, and 12, respectively (ICC=0.25). We intend to use the method of restricted maximum likelihood (REML) to fit a linear mixed model to the observations of KWC. The model will have random effects terms for hubs, key workers within hubs and assessments within key workers (within hubs), and fixed effects terms for time (0, 3, 6, and 9 months, which span the three 3-month periods) and condition (control or intervention). On the basis of 2000 simulations of the trial for each of the 4 scenarios, we find that the minimum effect sizes that can be detected with 80% power range from 0.49 to 0.68 (Table 2). For each of the 4 scenarios, the minimum detectable effect size was calculated from 2000 simulations of the trial in which restricted maximum likelihood was used, in each simulation, to estimate the variance components, and an F test was used to compare the predicted means. As a contingency against an overall system improvement over time, each scenario also included an underlying trend over time (an increase of 2 units in key worker confidence (KWC) from the first to the last assessment). Simulations assumed baseline KWC=70, total variance=18, and between-hubs variance=2 (11.1% of the total variance) with a corresponding coefficient of variation (CV)=6.1%. Simulations were also repeated with total variance=49 and between-hubs variance=5 (10.2% of the total variance) with a corresponding CV=10%. As expected, the minimum detectable effect sizes (not shown) were very similar—a consequence of standardizing the effect difference by the square root of the total variance. Accordingly, we conclude that this study has moderate-to-high power to detect moderate effect sizes for outcome variables that are measured repeatedly on key workers.

### Data Analysis

#### Quantitative Analysis

The repeated measurements (4 in total) on individuals in the 6 hubs over the three 3-month periods will be analyzed using linear mixed models. For measurement scales that are not too coarse (eg, more than 7 distinct values), the statistical analyses will make use of the REML algorithm to fit the mixed model. This algorithm will take account of missing (at-random) assessments and also allows the exploration of various autocorrelation models for the repeated measurements. Comparison of the *control* and *new program* means (adjusted for period effects) will be assessed with an *F* test. If required, after inspection of diagnostic plots of residuals and fitted values, an empirical logit transformation will be used to reanalyze any item scales that are bounded and coarse. Generalized linear mixed-model techniques will be used to analyze binomial or other categorical data. Cumulative effects of exposure to the new program on key outcome variables will also be explored using the same methods. The final analysis will be conducted after all participants have had adequate opportunity to be assessed at the end of the third period (ie, at 9 months), and the database has been locked. Process evaluation will assist in the assessment of factors that impact on the feasibility, success, and sustainability of the intervention strategies and new program. A detailed statistical analysis plan can be accessed by contacting the corresponding author.

**Table 2:** Minimum effect sizes.

Key workers per hub^a,b^	Intraclass correlation^c^	Minimum detectable effect size
6	0.25	0.68
6	0.5	0.56
8	0.25	0.59
8	0.5	0.49

^a^Effect size is the expected difference between the 2 conditions (intervention-control) divided by the SD (ie, the square root of the total variance).

^b^Detectable with 80% power for the proposed stepped-wedge design with 6 hubs, 3 periods, and 4 assessment time points.

^c^Intraclass correlation: between individual variance divided by the sum of the between and within individual variances.

#### Economic Analysis

The economic analysis includes a cost analysis, together with efficiency analyses based on either of the following: (1) simple *dominance* (intervention cheaper/outcomes better or no different) or *dominated* (intervention more expensive and outcomes same or worse) and (2) more complex analyses that examine cost in relation to different definitions of value.

Simple dominance will be established using cost-effectiveness analysis that compares program costs with physical outcomes (based on primary and secondary outcomes for parents and key workers collected in the trial). The cost analyses will be conducted to determine whether the capacity building program is cheaper or more expensive than current practice, primarily from the service provider perspective but also from the perspective of parents and the health care system.

If the costs of the capacity program are not cheaper and the program is more effective, then *value* has to be analyzed to establish *value-for-money*. One way is to list the range of primary outcomes and secondary outcomes that have policy meaning to stakeholders (so called *cost-consequences analysis*) and/or express these as a series of cost-effectiveness ratios (eg, *net cost per parent satisfaction score* and *net cost per unit of staff well-being score*). A judgment call could then be made by key staff as to whether these benefits constitute sufficient return on the investment.

Another way is to place a dollar value on these outcomes to establish a return on investment using cost-benefit analysis. There are various methods available to do this (eg, value of *statistical lives* where premature death is prevented; human capital methods; and scenario-based techniques such as willingness-to-pay or conjoint analysis). A decision on the most appropriate technique, if the trial yields this result, will be made in conjunction with the service provider senior management.

#### Qualitative Analysis

The focus groups will be recorded and transcribed verbatim. Qualitative data will be analyzed and coded using an inductive, thematic approach informed by grounded theory [57]. NVivo12 software will be used to manage the data during the analysis of results [58]. Similarities and differences will be compared within and across focus groups to identify emergent themes. This will be used to generate a conceptual analysis which will be aligned with strengths and gaps in current theory, evidence, and practice to increase understanding of key issues relating to the feasibility and impact of the intervention strategies.

## Results

This study was funded in October 2014 supported by an Australian National Health and Medical Research Council Partnership Project grant (Grant number 1076861) and cash and in-kind contribution from Yooralla as the associated partner organization. This is a multiyear study, and the final year comprises the trial outlined in this study. The trial is registered with the Australian New Zealand Clinical Trials Registry (ACTRN12617001530314), retrospectively registered on 3 November 2017. Results are expected to be available by the end of 2019.

## Discussion

### Overview

Strategies to improve the mental health and well-being of parents of children with a disability are urgently needed [59] and are timely given the current rollout of the National Disability Insurance Scheme (NDIS) in Australia. The NDIS aims to improve the lives of individuals with a disability and their families. To our knowledge, there are currently no evidence-based interventions delivered within an ECIS to directly support the mental health of parents and carers of children with a disability. However, supporting mental health as well as providing child-related input is a key factor on the pathway to enabling their own, as well as their child’s, full participation in society and their community and to achieve positive health outcomes [60-62].

This study will pilot an intervention program embedded in a capacity building framework to increase ECISs’ support for the mental health of parents of children with a disability. Providing professional development about the importance of recognizing and supporting parental distress, the implications on positive child development, and the importance of seeking support for their own well-being is novel to this key worker cohort. Evidence for the effectiveness and cost of this type of intervention is lacking in the literature but is urgently needed to further understand the best models of care by staff to achieve well-being outcomes for families and children with a disability. It is anticipated that the capacity of their key worker and thus their confidence and job satisfaction may lead to improvements in their own mental well-being. The training is specifically designed to build the capacity of key workers to understand when a parent is struggling and would like to be connected with additional supports and that they are supporting an at-risk population group for poor mental health. There is a strength in providing training to all key workers to be able to identify and assist parents in the first instance of becoming aware of any psychological stress or distress as the availability of a key worker with a psychology background on each team is subject to recruitment by the service. The trainings focus on providing resources to staff and helping them to identify when the parent’s needs are out of their scope, we anticipate, will raise their awareness of when they need to refer customers to external supports. The focus of the early childhood intervention service is to aid the development of the child and to empower the family so key workers do not provide ongoing support for parental mental health but rather refer as appropriate.

On the basis of the research findings that emerged from the exploratory phase of this research, it is imperative to intervene at not only the key worker level but also to introduce and trial strategies at the organizational level to achieve sustainable change for families accessing the service for their child. A partnership between academics, clinicians, and the delivery organization allowed the development of a program that was feasible and relevant to the service that could be embedded in the service delivery system and addressed a current gap in the literature. The study design is practical and ethical for research in this space, with each hub of staff and parents exposed to the intervention by the end of the trial. A knowledge translation plan is in place to embed successful components of the program into policy documents to support the ongoing accessibility of the program for current staff and upskilling of new staff. Successful components of the intervention will be made accessible online via the staff intranet for ease of access and to allow content to be updated as new evidence of best practice arises and external support details require updating. The participatory method taken to develop and deliver the intervention is ideal for the proposed sustainability of the program, with the potential to embed within the organization’s professional development schedule. The scalability of the program is possible for other disability organizations that support parents of a child with a disability, once the trial has assessed if the intervention produces significantly improved self-efficacy and mental health for staff and parents. In addition, if service improvements and increased parent satisfaction have been shown to occur, the service will be more closely aligned with the NDIS.

### Strengths and Limitations

A strength of this study is the stepped-wedged design, which allows the collection of mixed-method data, including quantitative data, also including economic data, and qualitative data from key workers and parents, which historically are a difficult cohort to involve in longitudinal research because of family demands. Particularly, the inclusion of the economic evaluation provides a model for future studies involving service delivery organizations. The design also addresses the ethical issue of conducting a blinded randomized control trial with a vulnerable population, as we deem it inequitable to provide care for mental well-being to only a cohort of parents accessing the service. A limitation of this study is that it is conducted within a changing policy and funding context, with the NDIS roll out underway in Australia, which is having an ongoing impact to service delivery within the partner organization and which we anticipate will be a potential confounder on the outcome measurements. For organizational reasons, the first 2 hubs are not randomly selected. This is not a major issue for assessment of the impact of the program on the partnering organization as we will use both this intrahub (each hub as its own control) as well as the interhub information when estimating the effect of the new program on the organization. Incomplete randomization does, however, impact on the external validity of the program. We seek to minimize potential bias by not revealing to the staff when the new program is scheduled to be allocated to their hub, although this may become self-evident in the last 2 hubs. Owing to this research being conducted in a real-world service delivery context, there is possible contamination because of a small number of key workers working across multiple sites that will receive the intervention at varying times which may not be overcome by the methods introduced to minimize contamination impact on results. Although methods to minimize the impact of this are in place, it is possible that training at 1 site will influence their service delivery approach to parents at their alternative work place that has not yet received the intervention and other staff may observe and learn, particularly because of the transdisciplinary approach. This will be addressed through discussions with relevant key workers who will be asked to avoid talking about their learnings with staff from hubs yet to receive the intervention to minimize contamination as much as possible. A further limitation of the research was that it was not feasible to have a third group that received only the well-being materials that are provided in conjunction with the face-to-face training to assess their impact independent of the impact of the training on parental well-being.

### Conclusions

This research will investigate a strategy to help break the negative cycle of poor mental health in parents of children with a disability that leads to poorer short and long-term outcomes for themselves, their child, and their family and their ability to contribute to the community and workforce. It is also urgently needed to provide staff training and clear work roles for the provision of support to parents to reduce worker stress, improve staff retention, and decrease stress for coworkers and families. Through building the knowledge and confidence of health professionals to support parents, it is likely that health professionals will feel increased self-efficacy and job-related well-being, which may also increase productivity and reduce job turnover. Furthermore, by improving the provision of mental health support for parents, it is likely children will benefit in terms of their own health, particularly mentally and socially, and their development because of the increased capacity of parents to care for their children.

## Appendix

Multimedia Appendix 1 Staff survey.

Multimedia Appendix 2 Parent survey.

Multimedia Appendix 3 NHMRC Partnership Project Grant Review Panel Report.

## Abbreviations

ECIS: Early Childhood Intervention Service
FCP: family-centered practice
KWC: key worker confidence
NDIS: National Disability Insurance Scheme
REDCap: Research Electronic Data Capture
REML: restricted maximum likelihood
VAS: visual analogue scale
WEMWBS: Warwick-Edinburgh Mental Wellbeing Scale
