# Supplementary material for: A Capacity Building Program to Improve the Self-Efficacy of Key Workers to Support the Well-Being of Parents of a Child With a Disability Accessing an Early Childhood Intervention Service: Protocol for a Stepped-Wedge Design Trial
Source: JMIR Res Protoc. 2019 Apr 3;8(4):e12531. doi: 10.2196/12531 (PMC6510062; doi:10.2196/12531)
Supplement: Multimedia Appendix 1 [file resprot_v8i4e12531_app1.pdf]

Section 3:
Please answer the following questions in regards to how confident are you at:

a) Understanding the challenges that parents face?

0 - Cannot do at all | 50 - Moderately can do | 100 - Highly certain can do

(Place a mark on the scale above)

b) Understanding the factors that impact on parents' wellbeing

0 - Cannot do at all | 50 - Moderately can do | 100 - Highly certain can do

(Place a mark on the scale above)

c) Understanding why parents' wellbeing may impact on their children

0 -Cannot do at all | 50 - Moderately can do | 100 - Highly certain can do

(Place a mark on the scale above)

d) Understanding how parents' wellbeing may impact on their child's progress in early intervention

0 - Cannot do at all | 50 - Moderately can do | 100 - Highly certain can do

(Place a mark on the scale above)

e) Talking to parents of children with a disability about their own wellbeing

0 - Cannot do at all | 50 - Moderately can do | 100 - Highly certain can do

(Place a mark on the scale above)

f) Knowing when parents are struggling with their wellbeing

0 - Cannot do at all | 50 - Moderately can do | 100 - Highly certain can do

(Place a mark on the scale above)

g) Knowing how to support parents' wellbeing

0 - Cannot do at all | 50 - Moderately can do | 100 - Highly certain can do

(Place a mark on the scale above)

h) Knowing how much time a Key Worker should spend on supporting parents' wellbeing

0 - Cannot do at all | 50 - Moderately can do | 100 - Highly certain can do

(Place a mark on the scale above)

i) Knowing when to refer parents for additional help

0 - Cannot do at all | 50 - Moderately can do | 100 - Highly certain can do

(Place a mark on the scale above)

j) Knowing where to refer parents to get mental health support

0 - Cannot do at all | 50 - Moderately can do | 100 - Highly certain can do

(Place a mark on the scale above)

Section 4:

The following are some questions about your job. Thinking about the past few weeks, how much of the time has your job made you feel each of the following?

	Never	Occasionally	Some of the time	Much of the time	Most of the time	All of the time
a) Tense	◯	◯	◯	◯	◯	◯
b) Uneasy	◯	◯	◯	◯	◯	◯
c) Worried	◯	◯	◯	◯	◯	◯
d) Calm	◯	◯	◯	◯	◯	◯
e) Contented	◯	◯	◯	◯	◯	◯
f) Relaxed	◯	◯	◯	◯	◯	◯
g) Depressed	◯	◯	◯	◯	◯	◯
h) Gloomy	◯	◯	◯	◯	◯	◯
i) Miserable	◯	◯	◯	◯	◯	◯
j) Cheerful	◯	◯	◯	◯	◯	◯
k) Enthusiastic	◯	◯	◯	◯	◯	◯
l) Optimistic	◯	◯	◯	◯	◯	◯

Section 5:

Please indicate how much you agree or disagree with the following statements

	Strongly Disagree	Disagree	Neither Agree nor Disagree	Agree	Strongly Agree
a) I can do my job well	○	○	○	○	○
b) I sometimes think I am not very competent at my job	○	○	○	○	○
c) I can deal with just about any problem in my job	○	○	○	○	○
d) I find my job quite difficult	○	○	○	○	○
e) I feel I am better than most people at tackling job difficulties	○	○	○	○	○
f) In my job I often have trouble coping	○	○	○	○	○
g) After I leave my work, I keep worrying about job problems I find it difficult to unwind at the end of a work day	○	○	○	○	○
h) I feel used up at the end of the work day	○	○	○	○	○
i) My job makes me feel quite exhausted by the end of a work-day	○	○	○	○	○

Section 6:
Below are some statements about your current supervisory support.
Please choose the response that best describes how you perceive the support received from your supervisor.

	Strongly Disagree	Disagree	Slightly Disagree	Neither Disagree or Agree	Slightly Agree	Agree	Strongly Agree
a) My supervisor strongly considers my goals and values	○	○	○	○	○	○	○
b) Help is available from my supervisor when I have a problem	○	○	○	○	○	○	○
c) My supervisor really cares about my wellbeing	○	○	○	○	○	○	○
