# Supplementary material for: A Capacity Building Program to Improve the Self-Efficacy of Key Workers to Support the Well-Being of Parents of a Child With a Disability Accessing an Early Childhood Intervention Service: Protocol for a Stepped-Wedge Design Trial
Source: JMIR Res Protoc. 2019 Apr 3;8(4):e12531. doi: 10.2196/12531 (PMC6510062; doi:10.2196/12531)
Supplement: Multimedia Appendix 2 [file resprot_v8i4e12531_app2.pdf]

Please indicate how much you agree the following statements over the last month?

	Strongly disagree	Disagree	Neither agree or disagree	Agree	Strongly agree
a) My keyworker has been interested in my social and emotional wellbeing	◯	◯	◯	◯	◯
b) My keyworker has supported my social and emotional wellbeing	◯	◯	◯	◯	◯
c) My keyworker has listened to my concerns	◯	◯	◯	◯	◯
d) My keyworker has been comfortable talking to me about how I am coping	◯	◯	◯	◯	◯
e) I am satisfied with the service I have received from Yooralla	◯	◯	◯	◯	◯
