# Supplementary material for: A Capacity Building Program to Improve the Self-Efficacy of Key Workers to Support the Well-Being of Parents of a Child With a Disability Accessing an Early Childhood Intervention Service: Protocol for a Stepped-Wedge Design Trial
Source: JMIR Res Protoc. 2019 Apr 3;8(4):e12531. doi: 10.2196/12531 (PMC6510062; doi:10.2196/12531)
Supplement: Multimedia Appendix 3 [file resprot_v8i4e12531_app3.pdf]

2013 NHMRC Partnership Projects Third Call for Funding Commencing 2014

Partnership Projects Grant Review Panel Report

**Application ID**	APP1076861
**CIA**	Professor Elizabeth Waters
**Application Title**	A health and wellbeing model of care for disability service providers

The following table summarises the assessment of your application against the Assessment Criteria. Scores are the PPGRP average. The Deemed Category score is calculated from the Weighted Assessment Criteria scores and determined by the Rating Range as stated in the Peer Review Guidelines.

Assessment Criteria	Score
Track records of the Chief Investigators relative to opportunity. (25%)	5.727
Scientific quality of the proposal and methodology. (25%)	5.455
Relevance and likelihood to influence health and research policy and practice. (25%)	5.182
Strength of the partnership. (25%)	5.364
**Weighted Score**	**5.432**
**Deemed Category**	**5**

1 Track records of the Chief Investigators relative to opportunity

The team has evidence of long-standing collaborative research and five are members who are recognized as experts internationally and/or nationally. The remainder are developing researchers. Track records of the majority are excellent and they bring together experiences in all aspects of the project.

2 Scientific quality of the proposal and methodology

The project's objectives to develop and evaluate a model are clear. The project is excellent in design and feasible in the four year time frame proposed. The application of a stepped-wedge cluster randomized control design to evaluate the model developed is appropriate. The evaluation will appropriately include process, impact, costs and outcome measures.

3 Relevance and likelihood to influence health and research policy

Given the roll out of the NDIS, and the importance of delivery of care to children in the community with disability either congenital or acquired, this project addresses an issue of considerable importance. Development and evaluation of a model that translates into optimal care delivery may impact policy if accepted by the government but their lack of engagement as a partner means that work will be required to translate the research into practice.

4 Strength of the partnership

The involvement of the partner is evident from the conceptual stages of the proposal to the final application, and shows that the project plan was developed collaboratively. The significant cash and in-kind contribution demonstrates commitment by the partner which has longstanding experience providing support services for people with disabilities.

5 (if applicable) Overall comments against the "Criteria for Health and Medical Research for Indigenous Australians"

N/A

6 Overall comments

A very good proposed project likely to be successfully achieved.
